# General Solvent-dependent Strategy toward Enhanced Oxygen Reduction Reaction in Graphene/Metal Oxide Nanohybrids: Effects of Nitrogen-containing Solvent

**DOI:** 10.1038/srep37174

**Published:** 2016-11-17

**Authors:** Wei-Yao Kao, Wei-Quan Chen, Yu-Hsiang Chiu, Yu-Hsuan Ho, Chun-Hu Chen

**Affiliations:** 1 Department of Chemistry, National Sun Yat-sen University, Kaohsiung 80424, Taiwan

## Abstract

A general solvent-dependent protocol directly influencing the oxygen reduction reaction (ORR) in metal oxide/graphene nanohybrids has been demonstrated. We conducted the two-step synthesis of cobalt oxide/N-doped graphene nanohybrids (CNG) with solvents of water, ethanol, and dimethylformamide (DMF), representing tree typical categories of aqueous, polar organic, and organic N-containing solvents commonly adopted for graphene nanocomposites preparation. The superior ORR performance of the DMF-hybrids can be attributed to the high nitrogen-doping, aggregation-free hybridization, and unique graphene porous structures. As DMF is the more effective N-source, the spectroscopic results support a catalytic nitrogenation potentially mediated by cobalt-DMF coordination complexes. The wide-distribution of porosity (covering micro-, meso-, to macro-pore) and micron-void assembly of graphene may further enhance the diffusion kinetics for ORR. As the results, CNG by DMF-synthesis exhibits the high ORR activities close to Pt/C (i.e. only 8 mV difference of half-wave potential with electron transfer number of 3.96) with the better durability in the alkaline condition. Additional graphene hybrids comprised of iron and manganese oxides also show the superior ORR activities by DMF-synthesis, confirming the general solvent-dependent protocol to achieve enhanced ORR activities.

Oxygen reduction reaction (ORR) is the key process that governs the performance of fuel cells and metal-air batteries, which are promising solutions for the great demand of clean energy^1^,^2^. Although Pt and its alloys are recognized as the most efficient ORR catalysts, their high production cost and short lifetime limit the opportunity of commercialization. Abundant, low-cost transition metal oxides with high chemical stability and catalytic activities are potential alternatives to Pt-related materials. Highly conductive, porous graphene materials hybridizing with transition metal oxides are promising inexpensive candidates with remarkable activities achieving four-electron-transfer ORR^3^,^4^,^5^,^6^,^7^,^8^,^9^,^10^.

Solvent molecules, in a typical liquid phase synthesis, are present in large excess and regulate interactions between reagents and environments. Large occupation portions of solvents are anticipated to lower kinetic barrier for specific functionalization and manipulate material formation^11^. Homogeneity of solvents also ensures a reliable, reproducible material preparation. Despite the great effort on synthesizing various graphene nanohybrids in different solvents, the systematic studies regarding solvent effects on the composite preparation and the ORR activities remain unexplored. With hydrophilic graphene oxide (GO) commonly used as starting materials for preparation, polar solvent environments (such as water and ethanol) are frequently reported. Dimethylformamide (DMF) that contains nitrogen and carbonyl groups is widely recognized as reducing solvent capable of inhibiting hydrolysis rates for nanomaterial formation^12^. Few reports proposed graphene functionalization assisted by DMF with limited mechanistic insights. These three solvents together with various combination ratios have been used for most of graphene/metal oxide synthesis. General guidelines regarding synthetic roles of the solvents in controlling ORR performance are greatly lacked.

Herein, we studied the solvent-dependent ORR activities of metal oxide/graphene nanohybrids prepared in solvents of water, ethanol, and DMF. As these solvents contain different terminal groups (hydroxyl, carbonyl, and amino ones), we observed the variation of nitrogenation, porosity, nanoparticle loading, and ORR activities controlled by solvent environments in the cobalt oxide/N-doped graphene hybrid (CNG). The DMF-synthesis enables the much improved N-doping (2.3-time greater), wide-range distributed porosity, and the unique micron-void assembly. On the basis of the spectroscopic results, the effective nitrogenation in DMF synthesis is potentially catalyzed by the DMF-coordinated cobalt complexes. Following these features, we generally observed the graphene hybrids of manganese and iron oxides by DMF-synthesis exhibiting the stronger ORR activities than these obtained from ethanol system, confirming the solvent-dependent strategy critical in producing highly ORR-active electrocatalysts.

## Methods

### Material synthesis

The concentration of the GO aqueous suspension used for the synthesis was 10 mg/mL^13^. To synthesize the cobalt oxide/N-doped graphene hybrids (CNG), we selected three different solvents of DMF, ethanol, and water for the synthesis. The GO suspension (0.635 mL) was first added in a desired solvent of 50 mL, followed by the addition of aqueous 0.2 M Co(Ac)_2_ (2.4 mL) and then 1 mL of NH_4_OH at room temperatures. The mixture was refluxed for 10 hours at 90 °C. Afterwards, the resultant was reacted in a 23 mL autoclave at 150 °C for 3 hours. Samples prepared by DMF, ethanol, and water are denoted as CNG-DMF, CNG-EtOH, and CNG-H_2_O, respectively. The products were cleaned by ethanol and centrifugation for several times, and dried in an oven overnight. For the preparation of manganese oxide/N-doped graphene (MNG) and iron oxide/N-doped graphene hybrids (FNG), the procedure is similar to that of CNG except the precursors were substituted with Mn(Ac)_2_ and Fe(Ac)_2_, respectively. The MNG products prepared by DMF and ethanol are denoted as MNG-DMF and MNG-EtOH, respectively. FNG samples by DMF and ethanol are named as FNG-DMF and FNG-EtOH as well. The control experiments following CNG procedure with the absence of cobalt acetate produced reduced-GO in three different solvents, denoted as rGO-DMF, rGO-EtOH, and rGO-H_2_O. In contrast, the control experiments following CNG-DMF without adding GO were also conducted (see Supporting information). The UV-vis study of DMF coordination was conducted by measuring a solution of 0.2 M Co(Ac)_2_ (2.4 mL) mixed with 50 mL of DMF.

### Characterization

Transmission electron microscope (TEM) images were obtained by Philips CM200 at 200 kV. Samples for SEM (scanning electron microscope) measurement were prepared by drop-casting onto silicon substrates, and the images were acquired by a Zeiss Supra 55 Gemini FE-SEM. The X-ray diffraction (XRD) patterns were acquired using D2 Phaser. The specific surfaces and nitrogen adsorption-desorption isotherms were collected measured by Micromeritics ASAP 2010. Infrared spectra (IR) were recorded on a Perkin Elmer 100 FTIR spectrometer. The data of X-ray photoelectron spectroscopy (XPS) were obtained by a Kratos Axis Ultra DLD with a Mg/Al achromatic source. The weight percents of graphene of the composites were determined by thermogravimetry analysis (TGA) on a PerkinElmer TGA4000 with a heating rate of 10 °C min^−1^.

### Electrochemical Measurements

For the electrode preparation, one mg of the CNG catalysts (and also Pt/C) were dispersed in 1 mL of ethanol under sonication for 15 min to obtain a homogeneous suspension. Then 10 μL of the suspension was loaded onto a glassy carbon electrode of 3 mm in diameter followed by a 10 μL drop of 0.1% nafion in ethanol. A homemade electrochemical cell was used to perform cyclic voltammetry (CHI 704E, CH Instrument) with a platinum wire (counter electrode), glassy carbon electrode (working electrode), and Ag/AgCl electrode (reference electrode). We also conducted the Pt-free electrochemical tests (Au as the counter electrode and Hg/HgO as the reference electrode) for all samples to make sure no Pt deposition on the testing samples during ORR (see Supporting information). The electrolyte was saturated with oxygen by bubbling O_2_ for 30 min before each run of the experiment. The results were collected after two test runs under a potential range between −0.6 to 0.1 V (vs. Ag/AgCl) at a scan rate of 5 mVs^−1^ to confirm a steady collection of data. The control experiments were conducted with N_2_ bubbling through the electrochemical cell in CV measurements.

## Results

### Synthesis and characterization

The CNG composites were prepared by the two-step procedure. The mixture of graphene oxide and Co(Ac)_2_ was refluxed in solvents first for 10 hours, then treated solvothermally at 150 °C to obtain the CNG products. The reflux time is critical to complete the interaction equilibrium of cobalt ions and graphene oxides. A maximized loading of cobalt species in the final graphene composites can be achieved after 10-hour reflux (see Fig. S-1). The second step of hydrothermal enables the formation of metal oxide nanocrystals on graphene sheets, as well as the reduction of graphene oxide^13^.

**Figure 1:**
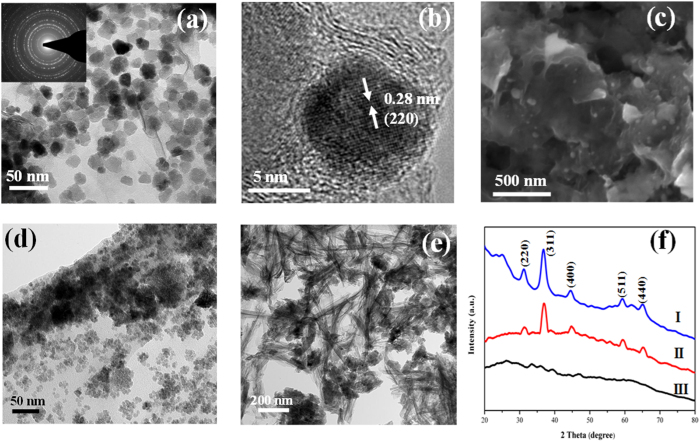
Characterization of CNG samples. (**a**) bright-field TEM images with the inset of electron diffraction patterns of CNG-DMF; (**b**) the high resolution TEM (HR-TEM) images of Co_3_O_4_ nanocrystals wrapped by graphene in CNG-DMF; (**c**) SEM images of CNG-DMF; TEM images of CNG-EtOH (**d**) and CNG-H_2_O (**e**); (**f**) the XRD patterns of CNG-H_2_O (I), CNG-EtOH (II), and CNG-DMF (III). The patterns of CNG-EtOH and CNG-H_2_O correspond to Co_3_O_4_ phase (JCPDS 9–418).

**Figure 2:**
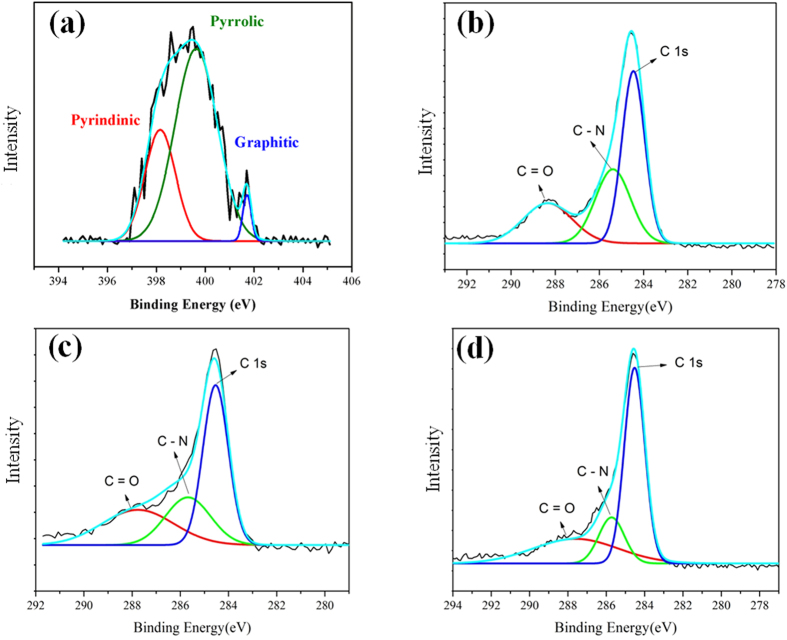
XPS spectra of CNG samples. (**a**) The N1s of CNG-DMF; the spectra of C1s of (**b**) CNG- DMF, (**c**) CNG-EtOH, and (**d**) CNG-H_2_O.

**Figure 3:**
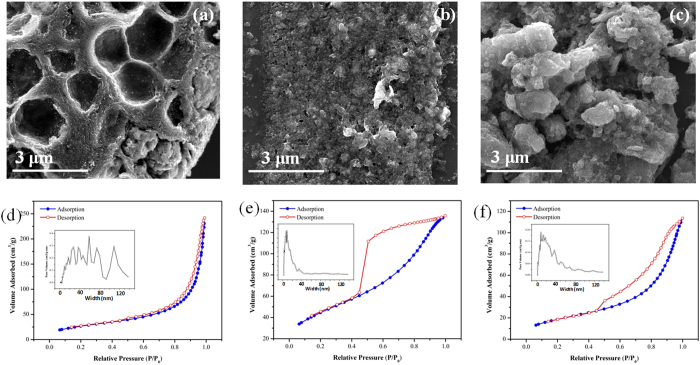
The morphological studies and N_2_ adsorption-desorption isothermal plots of CNG composites. The SEM images of (**a**) CNG-DMF (**b**), CNG-EtOH, and (**c**) CNG-H_2_O (all presented in the same scale). The N_2_ adsorption–desorption isothermal and pore size distribution plots (insets) of (**d**) CNG-DMF, (**e**) CNG-EtOH, and (**f**) CNG-H_2_O.

The TEM results of CNG-DMF show the round shape particles with diameters of 20.43 ± 3.78 nm distributed on graphene sheets without aggregation (Fig. 1a). The single-crystal nanoparticles with the lattice spacing of 0.28 nm corresponding to d_220_ in Co_3_O_4_ phase are encapsulated by few layers of graphene (Fig. 1b). The selected-area electron diffraction (SAED) patterns confirm the crystalline phase of Co_3_O_4_ (Figs 1a and S-2). The SEM images show that the nanoparticles in CNG-DMF decorate on graphene sheets with the moderate interval distance (Fig. 1c). For CNG-H_2_O and CNG-EtOH, their TEM and SEM images both demonstrate the highly dense aggregation of cobalt oxide nanoparticles on graphene sheets (Figs 1d,e and S-3). Particularly, one-dimensional features can be observed in CNG-H_2_O, quite different from the other two samples. The changes of solvent environments play critical roles in controls of morphologies and structural assembly of the composites. DMF environments potentially influence the hydrolysis and/or nucleation rates of cobalt oxides hybridizing with graphene^12^. Such the intensive nanoparticle aggregation in CNG-H_2_O and CNG-EtOH may hinder the diffusion kinetics for ORR.

The XRD results of CNG-DMF exhibit the very weak X-ray diffraction signals (Fig. 1f), compared to these of CNG-H_2_O and CNG-EtOH (Co_3_O_4_ phase). As the crystallinity of particles was characterized by SAED and HRTEM results, the weak XRD signals might be presumably due to the relatively poor crystallinity in the composites. DMF environment clearly affects the growth of cobalt oxide nanocrystals compared to that in water and ethanol^4^,^14^. The absence of graphene stacking peaks at 20–30 degree of two theta suggests the homogeneous hybridization between Co_3_O_4_ and graphene^4^.

As configurations and amounts of the doped nitrogen in graphene composites have critical impact on ORR activities^3^, we acquired X-ray photoelectron spectroscopy (XPS) data to monitor the configurations of nitrogen dopants. In Fig. 2a, the N 1 s deconvolution of CNG-DMF displays the presence of pyridinic (~398.1 eV), pyrrolic (399.6 eV), and graphitic (401.4 eV) nitrogen, all active for ORR^15^,^16^. The nitrogen contents (N1s) of the samples are summarized in Table 1, showing the highest nitrogen levels (7%) in CNG-DMF among these of CNG-H_2_O and CNG-EtOH (both ~3%). By removing NH_3_ from the CNG-DMF synthesis, we found a N-doping of 2–3%. As DMF is the only solvent containing nitrogen, the results suggest DMF acting as the additional N-source. The N-doping can also be identified by monitoring the formation of C-N bonding in XPS spectra at 285.4 eV of C 1 s (green curves in Fig. 2b–d)^17^. The quantity of C-N contents in CNG-DMF is higher than CNG-H_2_O and CNG-EtOH by factors of 2.4 and 2.2, respectively, corresponding well to the nitrogen contents in Table 1.

The SEM results with the same magnification reveal the morphological difference of graphene assembly between all the CNG composites (Fig. 3). CNG-DMF displays the three-dimensional micron-void assembly (Fig. 3a), where spherical (or particulate) assemblies of graphene are the main products observed in CNG-H_2_O and CNG-EtOH (Fig. 3b,c). The presence of micron-void graphene in CNG-DMF not only demonstrates the solvent effect, but also implies the tunable assemblies and porosities of graphene matrix. The results of BET surface areas and porosity studies are shown in Table 1 and Fig. 3d–f, respectively. Both CNG-H_2_O and CNG-EtOH comprise the characteristic of uniform mesopores associated with the well-defined capillary condensation step and hysteresis loops (type IV) at about 0.45 *P/P*_*0*_. CNG-DMF, however, has a relatively small hysteresis between the adsorption and desorption branches (at 0.45–0.9 *P/P*_*0*_), caused by no pore-blocking effect of narrow pores during desorption. Specific surface areas of CNG-DMF are measured to be 99 m^2^g^−1^ with a much wider range of pore size distribution, covering micro-, meso-, to macro-pores. Such the large pore sizes may reflect the less degree of graphene aggregation. The assembly behaviors affected by DMF are recognized by the unique porosity and micron-void structures above. Meanwhile, the alike pore size distribution (~10–20 nm) between CNG-H_2_O and CNG-EtOH suggests the similar structural assembly of graphene. Studies have shown that micro-, meso-, and macro-porosities all contribute to elevated ORR in different aspects^18^,^19^. The presence of wide-range mixture of porosity and micron-void structures in CNG-DMF could cover all the kinetic requirement and thus promote the accessibility of ORR catalytic sites^8^.

### ORR performance

**Figure 4:**
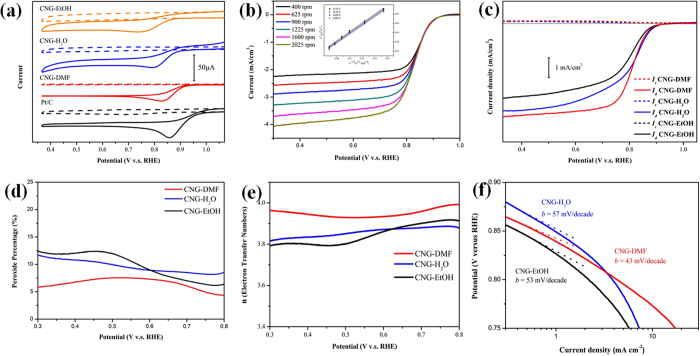
The ORR activities of CNG nanocomposites. (**a**) CVs of CNG samples and Pt/C in 0.1 M aqueous KOH with N_2_-saturated (dashed lines) and O_2_-saturated (solid lines) conditions. (**b**) LSVs of CNG-DMF in O_2_-saturated 0.1 M KOH at a scan rate of 5 mV/s with a range of rotation rates from 400 to 2025 (rpm). (**c**) The comparison of RRDE results of CNG samples at a rotation rate of 1600 rpm. I_r_ and I_d_ represent the ring and disk currents, respectively. The peroxide percentage (**d**), electron transfer numbers (**e**), and Tafel plots (**f**) of CNG composites.

To evaluate the ORR activities of the composites, the comparison of electrocatalytic activities in O_2_ and N_2_-saturated aqueous solutions (0.1 M KOH) is shown in Fig. 4a. CNG-DMF displays the superior activities with the largest ORR onset potentials at 0.980 V (cathodic current of 32 μA) to these of CNG-H_2_O (0.919 V) and CNG-EtOH samples (0.896 V). The onset potentials of CNG-DMF are close to Pt/C (1.005 V), better than the reported graphene/metal oxide composites mostly prepared with water and/or ethanol (Table 2)^3^,^20^,^21^,^22^,^23^. To measure the ORR kinetics of CNG-DMF, we acquired the rotating-disk electrode (RDE) results shown in Fig. 4b. A four electron transfer pathway could be involved as the RDE curves show a one-step reduction, instead of a two-step one, with the presence of limiting currents (0.3 to 0.7 V)^4^. The Koutecky-Levich (K-L) plots (inset of Fig. 4b) show an ideal linear relationship, suggesting the first-order reaction kinetics with stable numbers of electron transfer at the range of potentials^3^.

To further compare the ORR efficiency of the hybrid composites, we conducted rotating ring-disk electrode (RRDE) measurement to quantify the yield of peroxide species (Fig. 4c). In the diffusion region of 0.5–0.9 V (between the onset and saturated potentials), CNG-DMF has the highest current density and steepest slopes than CNG-EtOH and CNG-H_2_O, suggesting a rapid electron transfer process and high densities of active sites. The generation of ring currents can be observed at the corresponding onset potentials for the three CNG samples, where CNG-DMF shows the smallest ring current percents (~5.5%) than CNG-H_2_O (~10%) and CNG-EtOH (~11%) (Fig. 4d). The electron transfer numbers of CNG-DMF based on the RRDE results are 3.96, corresponding well to that of K-L plots^9^. CNG-H_2_O and CNG-EtOH have the decent electron transfer numbers of 3.85 and 3.80 respectively (at 0.3–0.7 V), but still lower than CNG-DMF (Fig. 4e). These results again emphasize the key role of solvent-dependent synthesis for achieving enhanced ORR activities.

**Figure 5:**
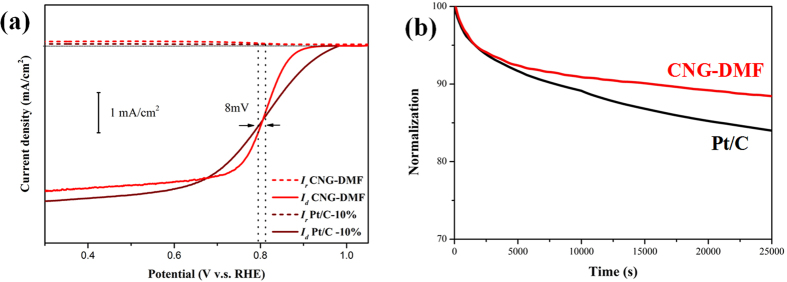
The LSV curves between CNG-DMF and Pt/C under 1600 rpm (**a**), and their durability tests in oxygen-saturated aqueous 0.1 M KOH (**b**).

The high ORR activities of CNG-DMF can also be recognized with the lowest Tafel slope of 43 (mV/decade) than CNG-H_2_O (57 mV/decade) and CNG-EtOH (53 mV/decade) in Fig. 4f ^24^. The RRDE comparison between CNG-DMF and Pt/C in Fig. 5a exhibits the relatively small difference of half-wave potential by 8 mV, suggesting their comparable ORR activities and diffusion kinetics. The durability tests show CNG-DMF with the higher amperometric stability than Pt/C (Fig. 5b). After 25000 seconds, CNG-DMF exhibits the relatively small decrease (9%) of current response, while the commercial Pt/C does a much greater current decay of 17%. According to the literature, the better current sustainability in CNG-DMF may relate to the graphene shell protection on cobalt oxide nanoparticles (Fig. 1b)^4^.

### Nitrogen doping study

Nitrogen-doping levels in graphene composites are critical for enhancing the ORR activities. To understand the N-doping efficiency via DMF and N-containing additive (i.e. NH_3_), we conducted additional CNG-EtOH and CNG-H_2_O samples with triple amount of ammonia addition. The nitrogen contents of the triple-ammonia CNG-EtOH and CNG-H_2_O are slightly increased by 1–2% compared to the original CNG-EtOH and CNG-H_2_O (Table 1), but not proportional to the added amount of ammonia, nor close to that of CNG-DMF (7.0%). These results indicate the limit of nitrogenation by following the conventional ammonia approach. The high N-contents in CNG-DMF is presumably caused by alternative mechanisms rather than solely by NH_3_ additive.

**Figure 6:**
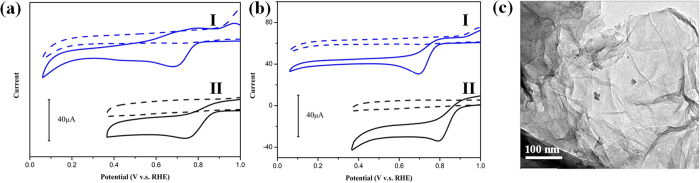
The effect of ammonia additive. (**a**) The CV comparison of triple-ammonia CNG-EtOH (labeled as I) and CNG-EtOH (labeled as II) with N_2_-saturated (dashed lines) and O_2_-saturated (solid lines) conditions. (**b**) The CVs of triple-ammonia CNG-H_2_O (I) and CNG-H_2_O (II). (**c**) The TEM images of the products following the CNG-DMF procedure without adding ammonia.

The triple-ammonia CNG-EtOH and CNG-H_2_O show the lower ORR activities with the decreased onset potentials of 0.808 V and 0.896 V respectively, compared to the regular CNG-EtOH and CNG-H_2_O (Fig. 6a,b). The fluctuated ORR activities suggest an unreliable N-doping by NH_3_ addition. DMF as the homogeneous solvent and N-source provide a stable synthetic environment for improved N-doping.

Although addition of excessive ammonia should be avoided, the presence of ammonia is necessary for the composite formation. The control experiments following CNG-DMF preparation without adding ammonia show very few cobalt oxide nanoparticles anchored on graphene sheets (Fig. 6c). Base NH_3_ is essential for the precipitation of cobalt hydroxide on graphene surface^3^,^25^.

**Figure 7:**
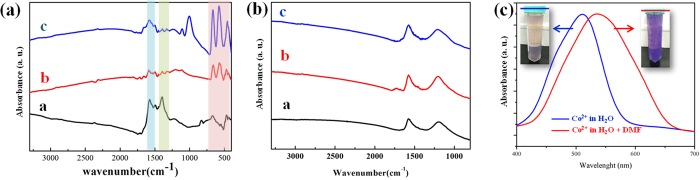
The functional groups and structural analysis. (**a**) The IR spectra of CNG-DMF, CNG-EtOH, and CNG-H_2_O, labeled by (**a**–**c**), respectively. (**b**) The IR spectra of the rGO-DMF, rGO-EtOH, and rGO-H_2_O (labeled by (**a**–**c**), respectively) without adding the cobalt oxide precursor. (**c**) The UV/Vis spectra of 0.2 M cobalt acetate in water (blue curve), and the red curve represents the addition of excess DMF in the 0.2 M cobalt acetate solutions shown in the blue curve. The insets are the solution photographs corresponding to the blue and red curves.

We also investigated the improved nitrogenation of CNG samples by infrared (IR) spectroscopy (Fig. 7a). The peaks in the range of 680 to 460 cm^−1^ (pink region) correspond to Co_3_O_4_ nanoparticles^26^, while these at 1575–1590 cm^−1^ are assigned to C = C bond (the blue region). The minor signals at ~1220 cm^−1^ can be due to C-OH and/or C-O-C peroxide, and signals at 1000 cm^−1^ are caused by oxidative carbon species (e.g. epoxides, carboxyls, dioxolane, etc.)^27^,^28^. In particular, the green region at 1400–1420 cm^−1^ corresponds to C-N bonding^27^,^28^,^29^. CNG-DMF shows the strong signals at 1403 cm^−1^, confirming the presence of C-N species. The control samples of CNG-DMF synthesis without mixing GO yield the Co_3_O_4_ nanoparticles with no nitrogen contents (Fig. S-5). Without adding cobalt acetate in three different solvents (following the two-step protocol of CNG synthesis), the resultants of rGO-DMF, rGO-H_2_O, and rGO-EtOH show the nearly identical spectra without the absence of C-N bonding (Fig. 7b). Under XPS analysis, these resultants display the similar nitrogen contents in the range of 2.4–3.0% (see Table 1), suggesting that the presence of cobalt cations is required for enhanced nitrogen doping in DMF synthesis.

## Discussion

Since DMF is the key source of nitrogen and needs cobalt cations for nitrogenation, the coordination of DMF bound to cobalt ions should be concerned. We compared the UV/Vis spectra of 0.2 M aqueous Co(OAc)_2_, acquired before and after mixing with excessive DMF (Fig. 7c). The blue shift of absorption λ_max_ and peak broadening caused by the DMF addition, as well as the color change (from pink to purple, insets of Fig. 7c), clearly demonstrate the presence of new DMF-coordinated cobalt complexes. The reported crystal structures reveal that the coordination of DMF to cobalt ions via carbonyl end is favored, rather than the ammine group^30^.

**Figure 8:**
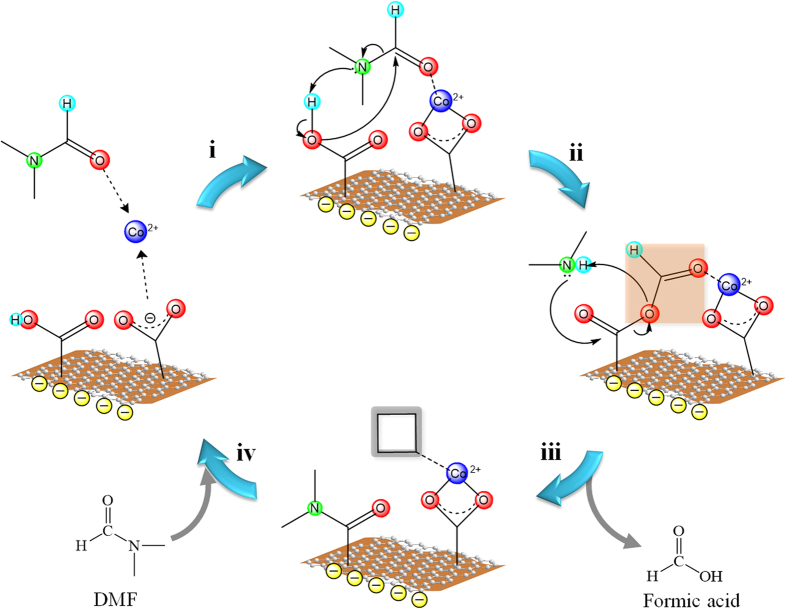
The proposed nitrogenation in CNG composites mediated by cobalt-DMF coordination complex. (**i**) The coordination of GO and DMF to cobalt cation forming a cobalt complex, (**ii**) the nucleophilic reaction between GO and DMF to release dimethylammine, (**iii**) the reaction between dimethylammine and carboxylic groups yielding amide, and releasing formic acid, (**iV**) the vacuum site available for DMF coordination for the next run of catalytic nitrogenation.

According to all the spectroscopic studies and experimental observation above, we propose the potential mechanism of catalytic nitrogenation mediated by the presence of cobalt-DMF coordination complexes (Fig. 8). Cobalt cations were first coordinated by the carboxyl groups in GO and DMF molecules with certain coordination geometry. The coordination of C = O (in DMF) to cobalt cation may increase the nucleophilic reactivities with the carboxylic groups of GO, and thus result in the release of dimethylammine. Subsequently, the dimethylammine may perform nucleophilic attack on the carboxylic groups on graphene to generate amide species and thus release formic acid. These amide species could be then converted as the doped nitrogen in graphene (pyrrolic, pyridinic, and graphitic forms) under the high pressure and temperature conditions, although the detailed transformation is not clear at this stage. We observed the decrease of pH values from ~10 (before the preparation) to 7 (after the preparation), presumably supporting the presence of formic acid as the side product. The IR signals of amide species additionally support the stage of amide formation. After the leaving of formic acid, the vacuumed coordination sites are available for more DMF molecules to carry out further nitrogenation.

The presence of cobalt coordination complexes may further provide the steric expansion to inhibit graphene sheet-to-sheet aggregation in the synthesis, based on the observed wide pore sizes and plateau currents. Such the expansion could favor the intercalation of cobalt oxide nanocrystals inside graphene stacks homogeneously, avoiding the sever aggregation of particles observed using nitrogen-free solvents (Fig. 1c v.s. S-3). In addition, studies have shown that thermal decomposition of DMF may release carbon monoxide^31^. The formation of CO microbubbles during the synthesis could potentially result in the micron voids, leading to that CNG-DMF is the only sample possessing micron-void features. Similar principle of utilizing microbubbles to form complex nanostructures (e.g. hollow spheres and double yolk-egg-like structures) has been successfully demonstrated^32^,^33^. The wide range of pore-size distribution in CNG-DMF could also be molded by the diverse diameters of CO microbubbles during graphene assembly.

CNG-DMF is the only sample that exhibits the appreciable plateau current (limiting current) as Pt/C does under RDE (Fig. 4c), giving the most efficient mass transport with the shortest kinetic-diffusion limit region. As a stable laminar flow over the electrode surface is closely associated with RDE kinetics, the highly dispersive cobalt oxide decoration and accessible porosity of CNG-DMF facilitate the ORR kinetics. The lack of plateau currents in CNG-EtOH and CNG-H_2_O may be attributed to (i) the rough surface geography caused by severe aggregation of nanoparticles, and (ii) the relatively narrow distribution of porosity.

To further study whether the electrocatalytic activities come from the N-doped graphene only or the hybridizing interfaces, we conducted the CV comparison of rGO-DMF and CNG-DMF. As shown in Fig. S-6, CNG-DMF has the superior ORR activities with more positive onset potentials and larger cathodic currents to rGO-DMF, revealing the synergistic ORR mainly attributed to the combination of N-doped graphene and cobalt oxide nanoparticles. The graphene contents determined by thermogravimetry analysis are 27%, 13%, and 19% for CNG-DMF, CNG-H_2_O, and CNG-EtOH, respectively (Table 1 and Fig. S-4), showing the lowest cobalt oxide loading in CNG-DMF. Such the low contents may possibly lead to the weak XRD signals, but no appreciable influence of Co_3_O_4_ crystallinity on ORR can be recognized as compared to the similar composites reported in the literature^3^. The interfaces comprised of metal oxides and graphene are commonly reported as the synergistic active sites^13^. However, the relatively high cobalt oxide contents in both CNG-H_2_O and CNG-EtOH do not show the better ORR activities than CNG-DMF. This indicates that the solvent-dependent features of nitrogenation, porosity, and particle decoration can be together more influential in manipulating ORR activities.

**Figure 9:**
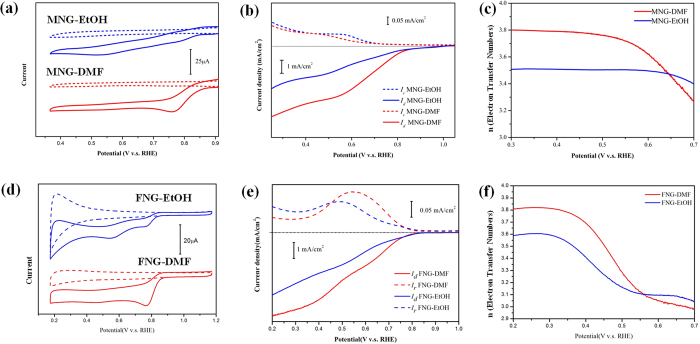
The comparison of ORR performance of MNG and FNG composites prepared in different solvents (DMF and ethanol). The CV comparison of MNG (**a**) and FNG composites (**c**) with N_2_-saturated (dashed lines) and O_2_-saturated (solid lines) conditions. The RRDE results of MNG (**b**) and FNG nanohybrids (**e**). The electron transfer numbers of MNG and FNG are shown in (**c**) and (**f**), respectively.

Based on the proposed mechanism, transition metals capable of forming coordination complexes with DMF could generally yield graphene composites that show improved ORR activities with DMF synthesis. We conducted additional preparation using manganese and iron cations due to their similar metal-DMF coordination behaviors to the cobalt system^34^,^35^. The resultants of manganese oxide/N-doped graphene (MNG) and iron oxide/N-doped graphene nanohybrids (FNG) were synthesized in DMF and ethanol for comparison (Fig. 9). The nitrogen contents (by XPS) of DMF-synthesis are generally higher than EtOH-synthesis by 80% for MNG and 50% for FNG. The XRD results show Mn_3_O_4_ and Fe_2_O_3_ phases for MNG and FNG, respectively (Figs S-7 and S-8). MNG-DMF exhibits the more positive onset potentials at 0.890 V with larger cathodic currents than MNG-EtOH (0.854 V). In addition, FNG-DMF shows the positive-shift ORR onset potential at 0.88 V with ~2.5-time greater current than FNG-EtOH (onset potential at 0.84 V). We observed the higher numbers of electron transfer of MNG-DMF (n = 3.8) than MNG-EtOH (n = 3.5) at 0.5 V, as well as for that in FNG-DMF (n = 3.8) higher than FNG-EtOH (n = 3.6) at 0.3 V (Fig. 9c and f). The ORR activities of FNG-DMF are even more efficient than some of the most promising graphene/iron oxide composites (Table 2)^4^. Both FNG-DMF and MNG-DMF show the better ORR performance than these in ethanol, confirming the generality of DMF-assisted ORR enhancement.

**Figure 10:**
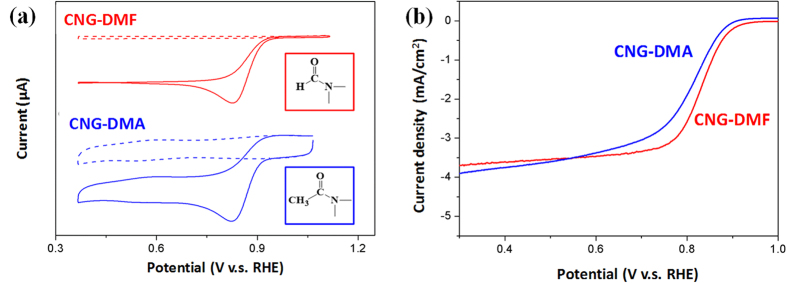
The ORR comparison of CNG composites prepared in different amide solvents. (**a**) The CVs acquired in 0.1 M KOH with N_2_-saturated (dashed lines) and O_2_-saturated (solid lines) conditions. The insets are molecular presentation of DMF (the red box) and DMA (the blue box). (**b**) The RDE results recorded at a rotation rate of 1600 rpm.

We further investigated whether amide solvents may have the similar role of ORR enhancement as DMF does. With the solvent criteria of high availability and reasonable cost, N,N-dimethylacetamide (DMA) was selected to produce the composites of CNG-DMA. The CV and LSV comparisons between CNG-DMF and CNG-DMA are made in Fig. 10. The onset and peak potentials of CNG-DMA are 0.96 V and 0.83 V (Fig. 10a), respectively, highly comparable to CNG-DMF. In the RDE profiles, CNG-DMA is also similar to CNG-DMF yet having the slightly more negative onset potentials (Fig. 10b). The similar functionality of amide solvents, consistent with the proposed mechanism, could be preliminarily identified. It is anticipated that amide-rich environments are effective and universal strategy to facilitate graphene nanocomposites achieving high ORR activities.

## Conclusion

We have revealed that, by simply changing synthetic solvents, much improved ORR activities can be achieved. The general solvent-dependent strategy of DMF synthesis enhances the ORR activities of graphene/metal oxide nanocomposites. Compared to the water and ethanol systems, the DMF synthesis enables the CNG products with unique micron-void assembly, wide-range porosity, aggregation-free decoration, and high nitrogen contents, responsible for the durable and efficient ORR performance. Greater loading amounts of cobalt oxides in the composites may not always be the main factor to interpret strong ORR activities. Although more advanced studies are required, the metal-DMF complexes on graphene basal plane are proposed to catalyze nitrogenation, as the results of improved diffusion kinetics and N-doping. This work may inspire a new perspective of solvent selection in preparing highly ORR-active graphene nanohybrids for the future clean energy applications. Detailed studies of the coordination mechanisms are actively pursuing.

## Additional Information

**How to cite this article**: Kao, W.-Y. *et al.* General Solvent-dependent Strategy toward Enhanced Oxygen Reduction Reaction in Graphene/Metal Oxide Nanohybrids: Effects of Nitrogen-containing Solvent. *Sci. Rep.* **6**, 37174; doi: 10.1038/srep37174 (2016).

**Publisher’s note:** Springer Nature remains neutral with regard to jurisdictional claims in published maps and institutional affiliations.

## Supplementary Material

Supplementary Information

## Figures and Tables

**Table 1:** The summary of graphene contents, nitrogen contents, and surface areas of CNG samples.

Samples	Graphene contents^a^	Nitrogen contents^b^	Surface areas (m^2^ g^−1^)
CNG-DMF	27%	7.0%	99
CNG-H_2_O	13%	3.1%	59
CNG-EtOH	19%	3.2%	160
rGO-DMF^c^	—	3.0%	—
rGO-H_2_O^c^	—	2.9%	—
rGO-EtOH^c^	—	2.4%	—

^a^The graphene contents are based on the TGA results under air.

^b^The atomic percentages of nitrogen are based on the XPS results.

^c^The control samples prepared by the two step protocol without adding cobalt precursors in different solvents.

**Table 2:** The comparison of ORR performance of the reported metal oxide/graphene catalysts.

Catalyst	Onset potentials	Current peak position	Reference
CNG-DMF	0.980 V (vs. RHE) −0.035 V (vs. Ag/AgCl)	0.828 V (vs. RHE) −0.1379 (vs Ag/AgCl)	This work
FNG-DMF	0.884 V (vs. RHE) −0.089 V (vs. Ag/AgCl)	0.767 (vs. RHE) −0.207 (vs. Ag/AgCl)	This work
C-Fe_3_O_4_	−0.187 V (vs. Ag/AgCl)	−0.340 V (vs. Ag/AgCl)	*Ref.* 22
Mn_3_O_4_/N-rmGO	−0.2 V (vs. Ag/AgCl)	−0.3 (vs. Ag/AgCl)	*Ref.* 21
Co_3_O_4_/N-rmGO	0.931 V (vs. RHE)	0.859 V (vs. RHE)	*Ref.* 3
NiCo_2_S_4_@N/S-rGO	−0.11 V (vs. Ag/AgCl)	−0.22 V (vs. Ag/AgCl)	*Ref.* 20
Fe_3_O_4_/N-GAs	−0.19 V(vs. Ag/AgCl)	−0.34 (vs. Ag/AgCl)	*Ref.* 4
rGFe-800a	−0.09(vs. Ag/AgCl)	−0.48 (vs. Ag/AgCl)	*Ref.* 23
