# Supplementary material for: General Solvent-dependent Strategy toward Enhanced Oxygen Reduction Reaction in Graphene/Metal Oxide Nanohybrids: Effects of Nitrogen-containing Solvent
Source: Sci Rep. 2016 Nov 17;6:37174. doi: 10.1038/srep37174 (PMC5112554; doi:10.1038/srep37174)

Supplementary Information for

General Solvent-dependent Strategy toward Enhanced Oxygen Reduction Reaction in Graphene/Metal Oxide Nanohybrids: Effects of Nitrogen-containing Solvent

Wei-Yao Kao, Wei-Quan Chen, Yu-Siang Ciou, Yu-Hsuan Ho, and Chun-Hu Chen*

Department of Chemistry, National Sun Yat-sen University, Kaohsiung, Taiwan 80424
E-mail: chunhu.chen@mail.nsysu.edu.tw

Figure S-1. The UV-vis spectra of the supernatant solutions, collected after the CNG-DMF synthesis, with varied reflux time. The peak at 530 nm corresponds to the absorption of cobalt species. After the 9-hr to 11-hr reflux, the cobalt concentrations in the supernatant are very low and reach equilibrium. Thus 10-hr reflux was selected to ensure the maximized and reproducible loadings of cobalt oxides in the CNG samples.

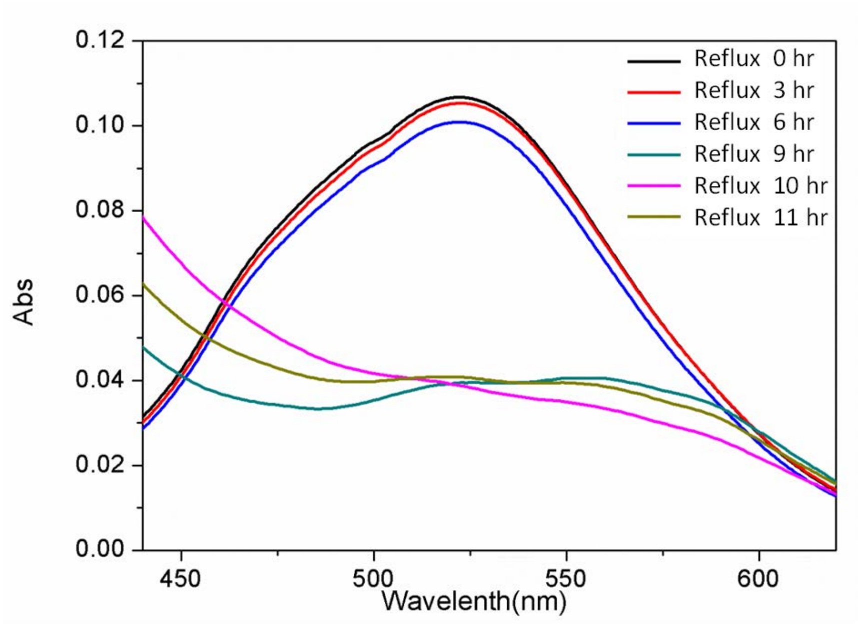

Figure S-2. The SAED patterns of CNG-DMF as shown in the inset of Fig. 1a. The pattern is indexed as spinel Co_3O_4.

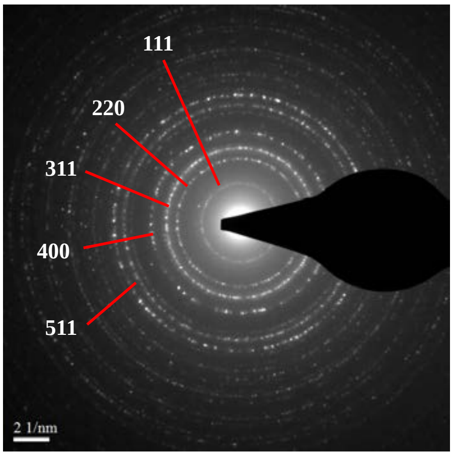

Figure S-3. The SEM images of the CNG composites: (a) CNG-EtOH and (b) CNG-H_2O

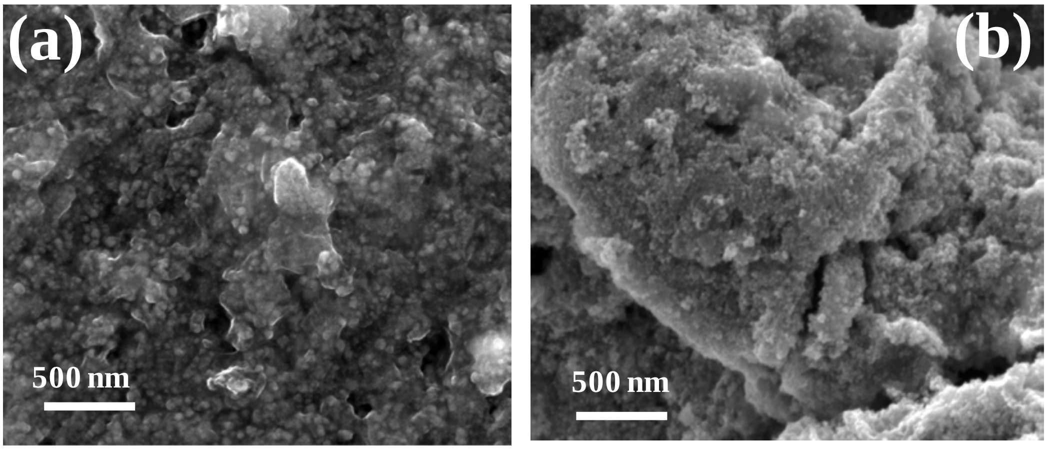

Figure S-4. The thermogravimetry analysis below shows the weight loss of a series of CNG samples. The weight losses for CNG-DMF, CNG-EtOH, and CNG-H_2O are 27%, 19%, and 13%, respectively.

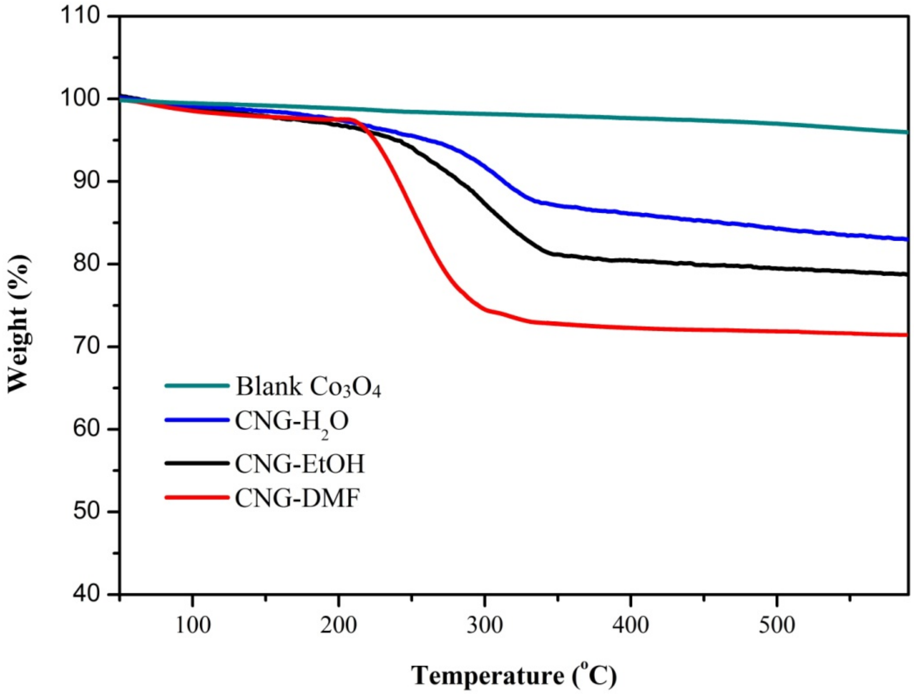

Figure S-5. The cobalt oxide products prepared by the control experiments following CNG-DMF synthesis without adding GO. (a) The XRD patterns show the Co_3O_4 phase (JCPDS 9-418). (b)-(c) The SEM and TEM images show that the particles are spherical in the diameters of ~20 nm. (d) The EDXS results under TEM show the nitrogen signals equivalent to the background noise levels, indicating no appreciable nitrogen present in the sample.

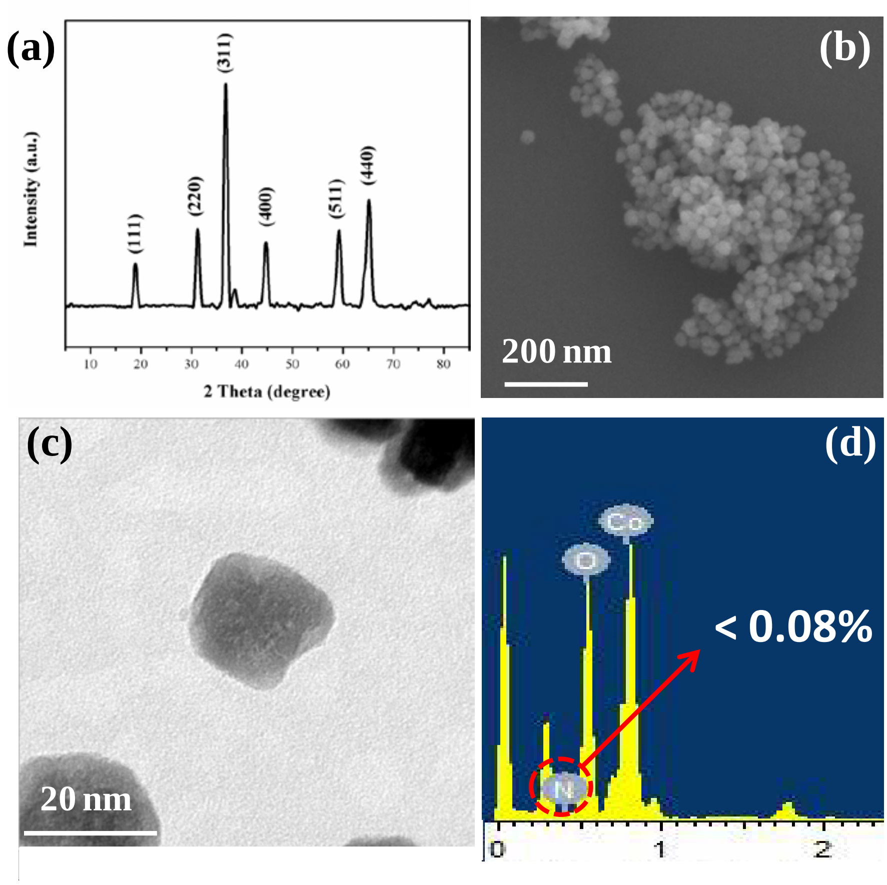

Figure S-6. The CV comparison of rGO-DMF and CNG-DMF with N_2-saturated (dashed lines) and O_2-saturated (solid lines) conditions. The onset potentials of rGO-DMF are observed at 0.87 V. The greater ORR activities of CNG-DMF than metal-free rGO-DMF demonstrate the synergistic ORR by the interfaces composed of the cobalt oxides and graphene.

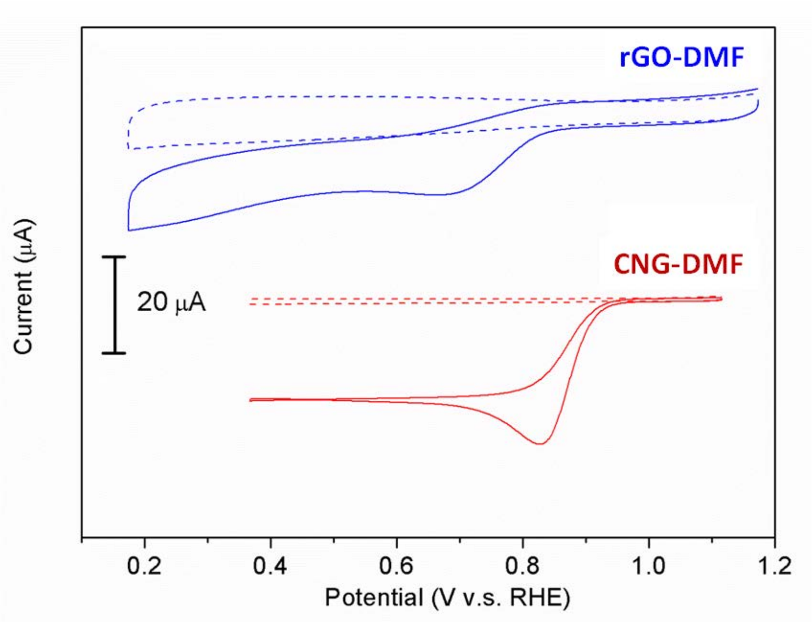

Figure S-7. XRD patterns of MNG samples. MNG-EtOH (A) and MNG-DMF (B) correspond to Mn_3O_4 phase (JCPDS 01-1127).

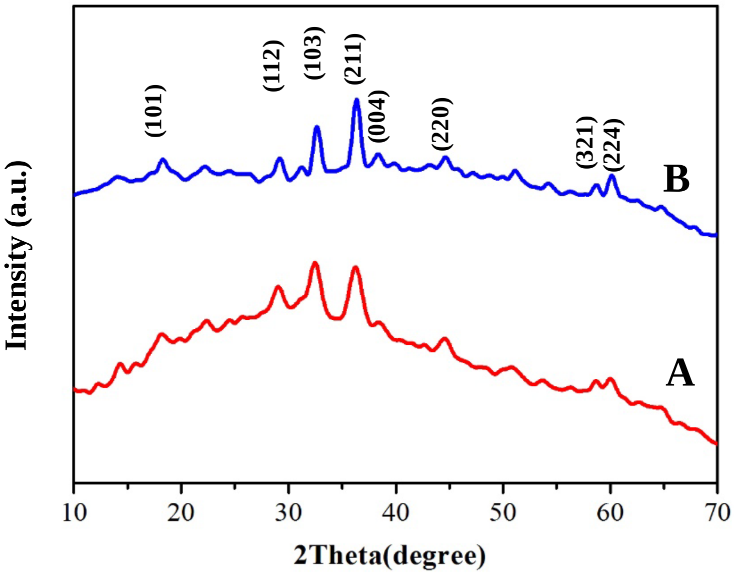

Figure S-8. XRD patterns of FNG samples. FNG-EtOH (A) and FNG-DMF (B) correspond to Fe_2O_3 phase (JCPDS 89-8104).

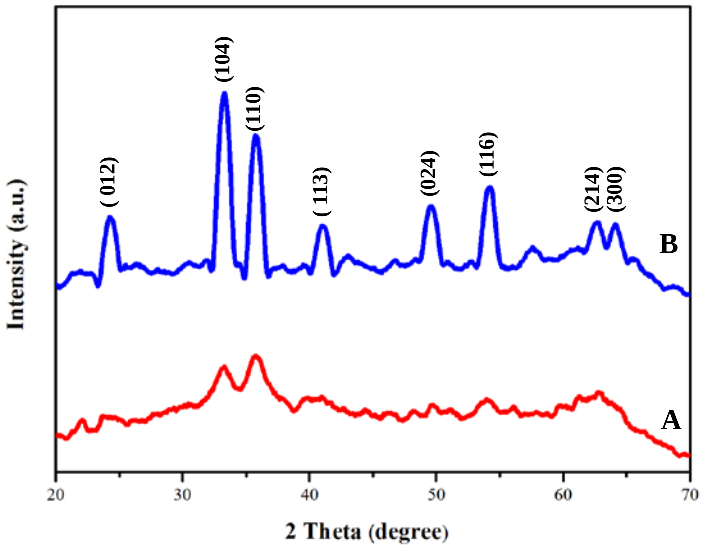

Figure S-9. The CV comparison of Pt-free and Pt systems with CNG-DMF (a), FNG-DMF (b), and MNG-DMF (c) is shown below (under O_2-saturated 0.1 M KOH). The Pt system is using Pt wires and Ag/AgCl as the counter and reference electrodes, respectively. In the Pt-free system, Au wires are the counter electrodes and Hg/HgO is the reference electrodes. After several repeating tests, all the samples show no significant electrochemical difference, indicating no Pt deposition on the CNG samples alternating their electrochemical nature.

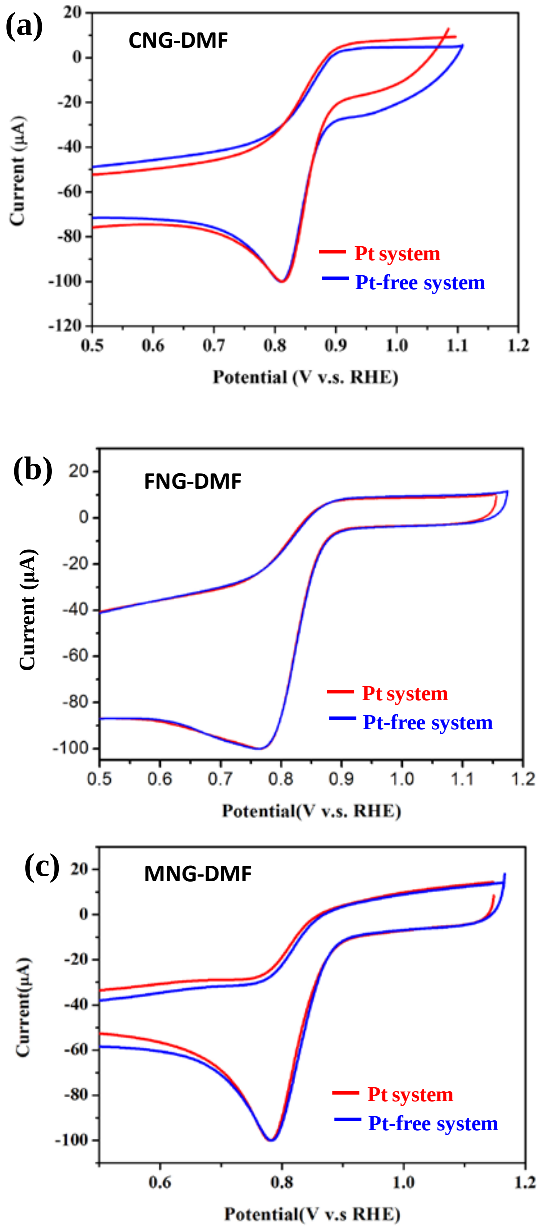

Supplement: Supplementary Information [file srep37174-s1.pdf]
